# Maintaining dosimetric quality when switching to a Monte Carlo dose engine for head and neck volumetric‐modulated arc therapy planning

**DOI:** 10.1002/acm2.13572

**Published:** 2022-02-25

**Authors:** Vladimir Feygelman, Kujtim Latifi, Mark Bowers, Kevin Greco, Eduardo G. Moros, Max Isacson, Agnes Angerud, Jimmy Caudell

**Affiliations:** ^1^ Department of Radiation Oncology Moffitt Cancer Center Tampa Florida USA; ^2^ RaySearch Laboratories AB Stockholm Sweden

**Keywords:** head and neck planning, Monte Carlo treatment planning, treatment planning algorithm transition

## Abstract

Head and neck cancers present challenges in radiation treatment planning due to the large number of critical structures near the target(s) and highly heterogeneous tissue composition. While Monte Carlo (MC) dose calculations currently offer the most accurate approximation of dose deposition in tissue, the switch to MC presents challenges in preserving the parameters of care. The differences in dose‐to‐tissue were widely discussed in the literature, but mostly in the context of recalculating the existing plans rather than reoptimizing with the MC dose engine. Also, the target dose homogeneity received less attention. We adhere to strict dose homogeneity objectives in clinical practice. In this study, we started with 21 clinical volumetric‐modulated arc therapy (VMAT) plans previously developed in Pinnacle treatment planning system. Those plans were recalculated “as is” with RayStation (RS) MC algorithm and then reoptimized in RS with both collapsed cone (CC) and MC algorithms. MC statistical uncertainty (0.3%) was selected carefully to balance the dose computation time (1–2 min) with the planning target volume (PTV) dose‐volume histogram (DVH) shape approaching that of a “noise‐free” calculation. When the hot spot in head and neck MC‐based treatment planning is defined as dose to 0.03 cc, it is exceedingly difficult to limit it to 105% of the prescription dose, as we were used to with the CC algorithm. The average hot spot after optimization and calculation with RS MC was statistically significantly higher compared to Pinnacle and RS CC algorithms by 1.2 and 1.0 %, respectively. The 95% confidence interval (CI) observed in this study suggests that in most cases a hot spot of ≤107% is achievable. Compared to the 95% CI for the previous clinical plans recalculated with RS MC “as is” (upper limit 108%), in real terms this result is at least as good or better than the historic plans.

## 1 INTRODUCTION

From the physics perspective, radiation treatment plan quality is a multifaceted concept encompassing calculated dose distribution metrics, the ability to deliver that dose accurately, and robustness with respect to movement.^1^ Accurate dose calculation is the foundation of the overall plan quality. Over the years, improvements in computing power and understanding of the underlying physics have led to increased calculational accuracy, particularly in the heterogeneous media. Switching from the model‐based algorithms (Type A by Knoos et al.^2^), such as the pencil beam, to Type B (e.g., superposition/convolution) allowed for better approximation of the dose on heterogeneous datasets by modeling the lateral spread of the energy deposition kernel. However, even the Type B algorithms cannot correctly predict the dose at the tissue interfaces. To do so, explicit modeling of the electron transport in the media is required. Hence, the most accurate approximation of the absorbed dose in the patient is provided by what can be called Type C algorithms, namely Mote Carlo (MC) simulations^3^ and the Grid‐Based Boltzmann Equation Solver (GBBS).^4^, ^5^ These algorithms natively report dose in tissue, which aligns with the emerging consensus.^6^, ^7^, ^8^ The clinical challenges of converting from the previous generation of algorithms to the current state‐of‐the‐art were enumerated in the American Association of Physicists in Medicine (AAPM) Report 105.^3^ While focusing on MC at the time, the report's conclusions, with the exception of statistical uncertainty considerations, are equally applicable to GBBS. One of the major concerns is reported dose to bone, which is reduced compared to Type B algorithms. Dose to air has no biological consequences per se, but may be important during optimization if the target encompasses an airway,^9^ and electronic disequilibrium can affect the mucosal dose.^10^ Precise treatment planning for head and neck (HN) cancers is particularly challenging not only because of the large number of critical normal structures in close proximity to the targets, but also due to the wide spectrum of inhomogeneities that can be encompassed by the target—from air to cortical bone. Thus, it is desirable to employ the most accurate dose calculation engine. A number of papers were dedicated specifically to the dosimetric effects of the Type C algorithms in the HN plans.^9^, ^10^, ^11^, ^12^, ^13^, ^14^, ^15^, ^16^, ^17^, ^18^, ^19^, ^20^ The dose differences were particularly large in bone,^15^, ^18^ although some authors also noted differences in air.^9^ The primary target dose heterogeneity often evaluated as *D*_2%_/*D*_98%_ in the target was typically higher for the Type C algorithms.^11^, ^13^, ^17^, ^18^ However, the comparisons were, as rule, done between the same plans *calculated* consecutively with the Type B and C algorithms,^13^, ^17^, ^18^ with the single exception of the study conducted by Kamaleldin et al.,^11^ where *reoptimized* plans were also evaluated.

In this study, we focus on the dosimetric aspects of making a switch from a specific Type B algorithm to Type C (MC). In particular, in our database of over 600 treated patients, the achievable clinical goal for the primary planning target volume (PTV) “hot spot” was ≤105% to a small volume of the target (*D*_0.03cc_). Given favorable complication rates,^21^ we were interested in duplicating the treatment approach as closely as possible, and therefore in ascertaining, among other things, what minimum hot spot is achievable when the planning is done with the commercial MC‐based algorithm.

## 2 METHODS

### 2.1 Overall strategy

It was pointed out in a number of publications that even if an ideal step‐wise PTV dose‐volume histogram (DVH) was achievable in optimization, it would be inevitably broadened or “blurred,” by the statistical noise in the final MC dose simulation.^22^, ^23^, ^24^, ^25^ Therefore, we first attempt to evaluate the effect of the statistical noise at the PTV DVH for an HN plan on a water‐equivalent patient‐shaped phantom and arrive at the optimal balance between computational time and accuracy. Second, it is known that one of the main differences between Type A or B and Type C algorithms is dose to bone and air. Both can be present in the PTV, albeit typically occupying a small proportion of its volume. Optimization is done primarily with a pencil beam algorithm (with the periodic full dose calculation), and subsequent full MC dose calculations degrade the dosimetric quality of the optimized plan due to the differences between the algorithms, which notably include the dose reporting media (water vs. tissue). To investigate this effect, we modified the pencil beam algorithm used in optimization, attempting to bring the calculated dose closer to dose‐to‐tissue. Finally, we compared the original clinical calculated dose distributions to those recalculated “as is” with RayStation (RS) MC, and to the results of our best efforts to reoptimize those plans in RS, using both Type B and C algorithms for the final dose calculation.

### 2.2 Dose calculation algorithms description

While the baseline clinical plans were developed in Pinnacle (Philips Medical Systems, Fitchburg, WI, USA) treatment planning system (TPS), the focus of this investigation is RS TPS (RaySearch Laboratories, Stockholm, Sweden). The final dose calculation in Pinnacle is performed with the collapsed cone (CC) superposition/convolution engine.^26^, ^27^ It propagates primary photon fluence in tissue, but the energy deposition kernel is calculated in water.^26^ As a result, the reported dose is in between tissue and water, but for simplicity is designated as dose‐to‐tissue, at least for relative densities close to 1.0.^6^ There are two final dose calculation options in RS: CC and MC. The former uses the formalism similar to Pinnacle but reports dose‐to‐water. The MC algorithm natively reports dose‐to‐medium (tissue).^28^ At present, neither algorithm is fast enough to be used in the optimization iterations. Both Pinnacle and RS use instead a fast pencil beam algorithm (singular value decomposition, or SVD^29^) for the vast majority of the optimization steps. The optimized SVD dose can be periodically corrected by the full dose calculation with the same algorithm as the final one. All final dose calculations were performed on a single NVIDIA Quadro P6000 GPU with the driver version 27.21.14.6109 (NVIDIA Corp, Santa Clara, CA, USA).

In addition to the commercial RS version (9B), we used the research build (10A‐DTK) that had an additional option for the optimization algorithm: the standard SVD engine was modified in an attempt to approximate dose to medium (DMSVD). To that end, for each voxel the SVD dose was multiplied by the medium‐to‐water ratio of mass energy absorption coefficients ${\left(\mu /\rho \right)}_{w}^{m}$.^30^

### 2.3 Effect of statistical uncertainty on the primary PTV DVH

Historically, while using a deterministic dose calculation algorithm, we have been evaluating the hot spot in the primary PTV as dose to a small volume (0.03 cc). It is still feasible to do with the MC algorithm but the statistical uncertainty has to be sufficiently small.^3^ The major areas of disagreement between the modern calculation algorithms, which generally model dose in water well, could be the dose in the inhomogeneous and/or buildup regions. Typical HN plans are potentially affected by both, as the PTV often approaches the skin and contains segments of bone and airways. We crop the PTV within 5 mm of the external contour, unless there is suspected superficial tumor involvement, in which case a bolus is used. It is, however, easy to eliminate the influence of the heterogeneity and build up in a model study, to focus solely on the statistical uncertainty. We assigned uniform water density to an entire patient dataset and added a 1.5 cm water bolus around it, so that the closest any point in the PTV approached the surface was 2 cm. A plan was optimized with the standard clinical objectives. In RS statistical uncertainty is defined, following the formulation by Kawrakow et al.,^31^ as one standard deviation averaged for all voxels receiving at least 50% of the maximum dose. The same plan was consecutively calculated with 1.0, 0.5, 0.3, and 0.1% statistical uncertainties. The resulting PTV DVH curves were plotted and the dose to the 0.03 cc hot spot was extracted.

### 2.4 Realistic treatment plans

The primary focus of this investigation was the comparison of the realistic treatment plans. Twenty‐one plans previously used at our institution to treat oro‐pharyngolaryngeal cancer formed the basis of this work, performed under a local, exempt retrospective study protocol. The original consecutive patient plans were developed for a single TrueBeam linear accelerator with a standard 120‐leaf Millennium multileaf collimator (MLC) (Varian Medical Systems, Palo Alto, CA, USA) in Pinnacle treatment planning system (TPS) v. 9.8 (Philips Medical Systems, Fitchburg, WI, USA). All calculations were performed on a 3 mm isotropic dose grid. All patients were planned with two full 6MV volumetric‐modulated arc therapy (VMAT) arcs using 4° control point spacing. These planning parameters were preserved for all subsequent dose recalculations. The original Pinnacle plans were designated as Pinn‐CC and evaluated without any modifications. Then the final dose was recalculated for the plans exported from Pinnacle without any changes (“as is”) with the RS MC algorithm (MC‐Recalc). After that, the plans were reoptimized from scratch in RS using the SVD optimization engine and CC final dose algorithm (RS‐CC). Finally, they were similarly replanned using the SVD or DMSVD optimization and MC final dose calculation (SVD‐MC and DMSVD‐MC, respectively). All plans treated the primary target (PTV_High) and the elective nodal volumes (PTV_Low) simultaneously and always contained two dose levels. The PTV_High/Low total doses for 17 cases were 60/48 (11) and 70/56 (6) Gy. For the remaining four cases, the PTV_High dose range was 60–68 Gy with the PTV_Low range of 50–54.4 Gy. The plans were always normalized so that at least 95% of each PTV's volume received its prescription dose. Additional requirements were to cover each gross tumor volume (GTV) volume minus 0.03 cc by the prescription dose and each PTV volume minus 0.03 cc by at least 95% of the respective *Rx* (PTV *D_V_*_‐0.03cc_ ≥ 0.95*Rx* [%]). The hot spot objective was to limit the maximum PTV_High dose to 105% of *Rx* (PTV_High *D*_0.03cc_ ≤ 1.05 *Rx* [%]).

The proxy for target dose inhomogeneity was the maximum dose to the PTV_High (*D*_0.03cc_). The PTV_High and PTV_Low minimum dose (*D_V_*_‐0.03cc_ ) was also recorded. The prescription dose conformality was quantified by the relative PTV_High volume of regret (VoR_100%_), defined as the ratio of the prescription isodose volume outside of the PTV_High to the PTV_High volume. The organs at risk (OARs) were evaluated as a group following the combined plan quality metric (PQM) methodology outlined by Gintz et al.^32^ In brief, the individual scores for the OARs present in the plan are summed up and renormalized to the maximum sum possible. The score functions are linearly continuous in the respective clinically relevant dose ranges. The detailed description of those individual OAR score functions can be found in the Supporting Information. The target dosimetric indices were extracted in ProKnow software (Elekta Inc., St. Charles, MO, USA)^33^ while PlanIQ (Sun Nuclear Corp., Melbourne, FL, USA)^32^ was used for the OARs, making comparisons independent of the original TPS. Both programs use similar fine dose and contour interpolation at the region of interest (ROI) edges, optimizing agreement with analytically derived DVH models.^34^

The target dosimetric indices described above were compared with repeated measures one‐way analysis of variance (ANOVA) corrected for multiple comparisons using statistical hypothesis testing (Tukey's method), implemented in GraphPad Prism v.9 (GraphPad Software, San Diego, CA, USA).^35^ The overall ANOVA test was followed by multiple comparisons between all columns (corresponding to different dose engines). The ANOVA test is known to be fairly robust with respect to deviations from the normal distributions of the samples. On the other hand, the OAR doses are prescription dose dependent and a priori come from a multimodal distribution. Therefore, the normal distribution assumption is presumably invalid and the best choice for statistical analysis was a nonparametric test, namely Friedman's test with Dunn's corrections for multiple comparisons, again followed by comparisons for each pair of columns.^35^ Throughout this study, the differences were considered statistically significant if *p‐*values were under 0.05.

## 3 RESULTS

### 3.1 Statistical uncertainty

**FIGURE 1:**
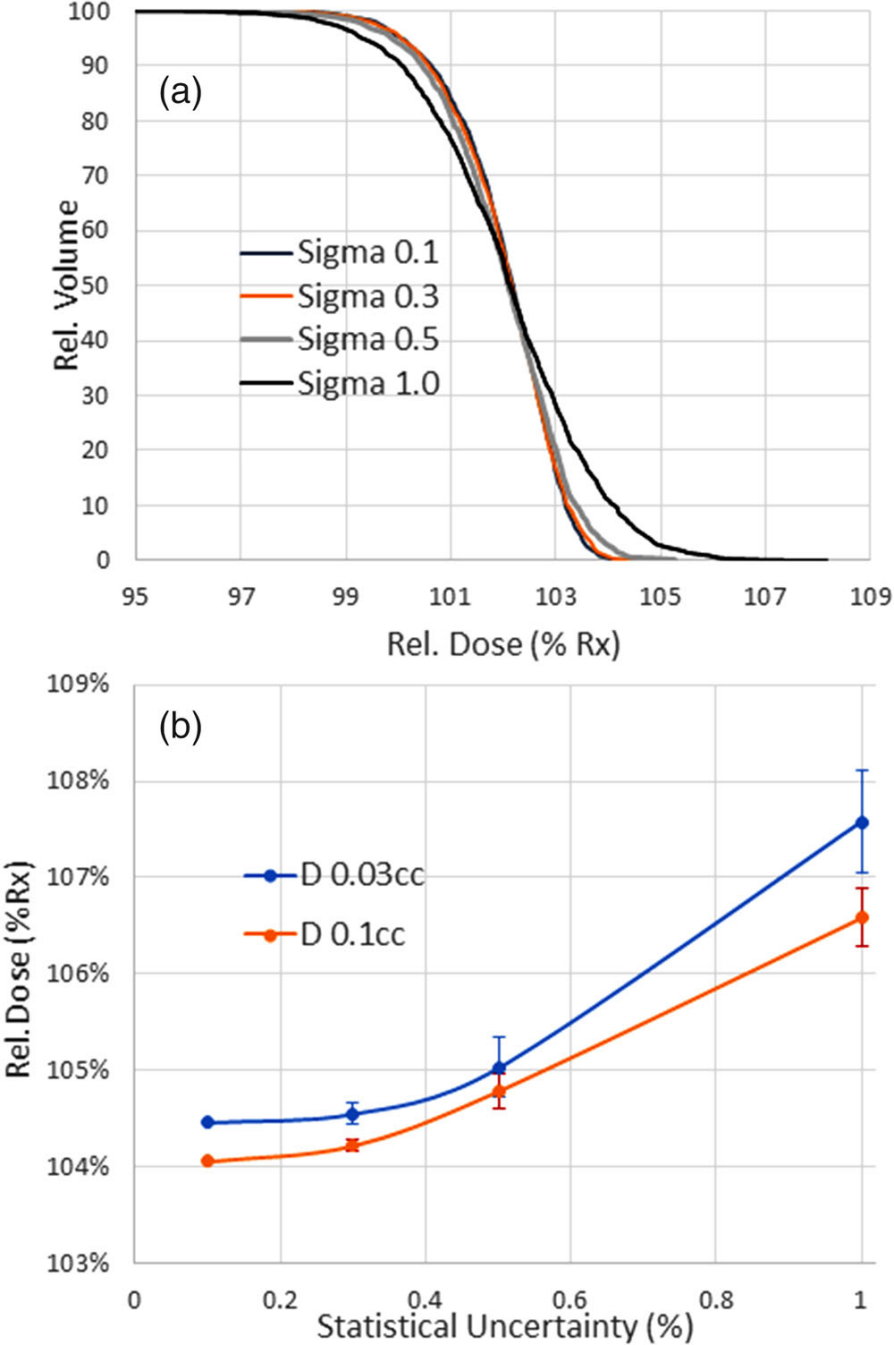
(a) Dose‐volume histogram (DVH) broadening with increased statistical uncertainty in Monte Carlo (MC) simulation of a head and neck (HN) plan as a function of calculation uncertainty. (b) *D*_0.03cc_ and *D*_0.1cc_ extracted from the DVHs above and plotted against calculation uncertainty. The error bars are corresponding calculation uncertainties

A family of the primary PTV DVH curves for an HN plan calculated on a homogeneous patient‐shaped dataset with varying statistical uncertainty is presented in Figure 1a. The extracted values of *D*_0.03cc_ are graphed in Figure 1b, with *D*_0.1cc_ shown for comparison. The difference in *D*_0.03cc_ between *σ* = 0.3% and *σ* = 0.1% is only ∼0.1%. The 0.3% relative statistical uncertainty is quite practical with the GPU‐accelerated RS MC simulations and it has become our standard for this work and subsequent clinical practice. All subsequent plans were calculated with this uncertainty value. The MC final dose simulation times ranged between 46 and 134 s for the cases with the smallest and largest primary PTVs (37 and 709 cm^3^, respectively). For comparison, increasing simulation uncertainty to 1% or 2% would lead to calculation times of 16 or 14 s for the smaller target and 30 or 21 s for the larger one. A deterministic RS CC calculations for similar plans would take 1524 s. The increase in dose computation time with 0.3% uncertainty, as expected, is appreciable in relative terms. However, 1 or 2 min for a full dose calculation in practice is almost negligible, as those are few and far between and constitute a small fraction of the overall optimization time.

### 3.2 Realistic treatment plans

As the first check, the average ratio (*n* = 21) of the mean PTV_High doses between the original Pinn‐CC plan and the same plans *recalculated* “as is” with MC (MC‐Recalc) was 0.997 ± 0.005 (1 SD), demonstrating general continuity between the planning tools. For the same plans, the mean dose (*D*_mean_) and the dose to 98% (*D*_98%_) of the intersection of the mandible with the primary target volume (Mandible ⋂ PTV_High) are presented in Table 1 for 14 plans where such structures existed. The average *D*_mean_ and *D*_98%_ values for the Pinnacle CC plans were higher compared to RS MC by 2.4 and 3.5%, respectively. Those differences were statistically significant (*p* < 0.0001).

**TABLE 1:** Analysis of variance (ANOVA) test of the Mandible ⋂ PTV_High *D*_mean_ and *D*_98%_ for the Pinnacle collapsed cone (CC) plans and the same plans recalculated with Monte Carlo (MC) in RayStation

	Dose metric (% of *Rx*)			
	*D*_mean_		*D*_98%_	
Descriptive statistics (*n* = 14)	Pinn‐CC	MC‐Recalc	Pinn‐CC	MC‐Recalc
Median	101.3	98.9	99.7	96.0
Mean	101.4	99.0	99.6	96.1
SD	0.58	0.89	0.82	0.61
Lower 95% CI	101.0	98.5	99.1	95.8
Upper 95% CI	101.7	99.6	100.0	96.5

	*p*‐values			
*D*_mean_	<0.0001			
*D*_98%_			<0.0001	

Abbreviation: CI, confidence interval.

Switching to the plans *reoptimized* in RS, the PTV_High maximum dose *D*_0.03cc_ was overall statistically significantly different (*p* < 0.0001) between the various algorithms (Table 2). In the subsequent pair‐wise analysis, the Pinn‐CC and RS‐CC averages were not statistically significantly different. The MC algorithm compared to Pinnacle CC resulted in the statistically significantly higher *D*_0.03cc_ on average. The DMSVD optimization engine provided no apparent improvement.

**TABLE 2:** Analysis of variance (ANOVA) test of the relative volume of regret (VoR_100%_) for PTV_High computed with different algorithms

	VoR_100%_				
Descriptive statistics (*n* = 21)	Pinn‐CC	MC‐Recalc	RS‐CC	SVD‐MC	DMSVD‐MC
Median	0.230	0.180	0.250	0.300	0.320
Mean	0.231	0.217	0.250	0.342	0.377
SD	0.075	0.098	0.071	0.151	0.167
Lower 95% CI	0.197	0.172	0.217	0.273	0.301
Upper 95% CI	0.266	0.261	0.282	0.411	0.453

	Multiple pair‐wise comparisons *p*‐values				
MC‐Recalc	0.9856				
RS‐CC	0.9694	0.7807			
SVD‐MC	0.0021	0.0003	0.0152		
DMSVD‐MC	<0.0001	<0.0001	0.0003	0.7336	

Abbreviations: CC, collapsed cone; CI, confidence interval; MC, Monte Carlo; RS, RayStation; SVD, singular value decomposition.

The overall ANOVA analysis showed statistically significant differences in the VoR_100%_ index (*p* < 0.0001). Both Pinn‐CC and RS‐CC resulted in more conformal dose distributions compared to MC, with the VoR being statistically significantly lower for the former two in the pair‐wise analysis (Table 2).

There was an observed corresponding trend in OAR sparing being slightly better with the CC‐based algorithms versus MC, but the differences were not statistically significant (Table 3).

**TABLE 3:** Nonparametric analysis of variance (ANOVA) (Friedman's) test of the organ at risk (OAR) plan quality metric (PQM) scores

	PQM score (%)				
Descriptive statistics (*n* = 21)	Pinn‐CC	MC‐Recalc	RS‐CC	SVD‐MC	DMSVD‐MC
Median	71.4	71.9	73.00	67.0	67.5
Mean	66.4	66.6	66.4	63.8	62.9
SD	17.1	17.0	17.3	18.5	19.1
Lower 95% CI	58.6	58.8	58.6	55.4	54.2
Upper 95% CI	74.2	74.3	74.3	72.2	71.6

	Multiple pair‐wise comparisons *p*‐values				
MC‐Recalc	>0.9999				
RS‐CC	>0.9999	>0.9999			
SVD‐MC	0.2183	0.0629	0.0541		
DMSVD‐MC	0.0248	0.0053	0.0044	>0.9999	

Abbreviations: CC, collapsed cone; CI, confidence interval; MC, Monte Carlo; RS, RayStation; SVD, singular value decomposition.

There were no statistically significant differences observed in the pair‐wise comparisons for PTV_High or PTV_Low minimum dose *D_V_*_‐0.03cc_ between Pinn‐CC, RS‐CC, and MC. This is not surprising, given that all plans were normalized to ensure the minimum target coverage.

## 4 DISCUSSION

We endeavored to commission the RS MC algorithm for HN planning while attempting to maintain continuity in planning goals as much as possible. A standard set of RS verification tests has been published,^36^ demonstrating agreement with measurements in both homogeneous and heterogeneous phantoms on par with similar commercial algorithms.^5^, ^37^, ^38^ Both Pinnacle and RS commissioning at our institution included a successful irradiation of the Imaging and Radiation Oncology Core (IROC) Houston HN end‐to‐end test phantom.^39^

A number of studies closely examined the effects of using Type C dose algorithms in the HN plans.^9^, ^10^, ^11^, ^12^, ^13^, ^14^, ^15^, ^16^, ^17^, ^18^, ^19^, ^20^, ^40^ The common thread among the findings is reduced dose to bone, when dose‐to‐tissue is reported, compared to the Type A or B algorithms. In our study, the average dose to the PTV_High (comprised primarily of soft tissue) was essentially indistinguishable between the Pinnacle CC and RS MC calculations for the same monitor units (MUs) and control point sequences. In the same plans, the dose to bone was reduced with the MC calculations compared to Pinnacle CC (Table 1). In our case the differences (negligible for soft tissue, 3.5% for Mandible ⋂ PTV_High) are less compared to the detailed analysis of the HN plans by Hardcastle et al.^15^ (1% for soft tissue and 5% for Mandible ⋂ PTV_High). One likely reason is that our historical clinical algorithm, Pinnacle CC, uses interpolated mass energy absorption coefficients for propagating TERMA in biological tissue and reports dose values between dose‐to‐water and dose‐to‐tissue.^6^ This is in contrast to the analytical anisotropic algorithm (AAA) used as the baseline by Hardcastle et al.,^15^ which inherently reports dose‐to‐water.^6^ A small error can also be hidden in the differences in beam models in the two TPSs.

Prior to acquiring RS, we accumulated a database of over 800 patients treated with radiation for HN cancers. All were planned with Pinnacle CC algorithm. We compared the rates of reactive placement of, and long‐term dependence on, percutaneous endoscopic gastrostomy (PEG) tubes in our series^21^ to other published outcomes. While not coming from a formal study with properly matched patient cohorts and statistical analysis, the data nevertheless hint that our complication rates might be lower than in the pooled photon chemoradiation analysis^41^ and comparable to the protons.^42^ Our PTV_High dose homogeneity requirements were more stringent than in published cooperative groups and institutional protocols^43^, ^44^, ^45^, ^46^: the goal for the maximum dose *D*_0.03cc_ was ≤105% of the prescription dose. There are no data to definitively link the favorable complications rates to this stringently controlled hot spot magnitude. However, such link is plausible and cannot be discounted a priori. Therefore, in the absence of convincing evidence to the contrary, it was imperative to make every effort to ensure practice continuation after switching to another TPS/algorithm. Since the original Pinnacle plans were calculated on a 3 mm isotropic dose grid, the present study used the same voxel size for continuity. It is close to the theoretical upper limit of 2.5 mm^47^, ^48^ and is adequate for the 5 mm MLC used in this study. If a smaller MLC such as 2.5 mm Millennium HD were used, a smaller grid size might be advisable. It quickly became apparent that it was impossible to maintain the 105% calculated hot spot when using RS with MC. This was confirmed in this study showing statistically significant difference in achievable PTV_High hot spot between the CC and MC algorithms (Table 4). The mean *D*_0.03cc_ value is 1.2% and 1.0% higher for RaySearch MC compared to Pinnacle and RaySearch CC, respectively. The rigorous analysis of the influence of MC statistical uncertainty on the DVH “blurring” is quite involved, and so is removing of this uncertainty, or “deconvolving” the DVH to arrive at the noise‐free curve.^22^, ^24^ However, an estimate can be made by simulating a family of the DVH curves for the same plan simulated with the progressively reduced statistical uncertainty.^49^ In its limit, the solution converges to the *real* DVH with zero uncertainty.^50^, ^51^ Previously, the value of 0.15% was suggested as the relative statistical uncertainty for the target DVH curve to be considered effectively noiseless.^49^ We used the 0.1% value, compared to 0.3% uncertainty used for planning. Based on that assumption, only a very small portion of the increase of the hot spot dose in the MC‐based calculations with 0.3% uncertainty, compared to the deterministic CC‐based algorithms, can be attributed to the statistical “blurring” of the PTV DVH (Figure 1). Thus, an approximately 1% overall average difference needs an alternative explanation. The natural assumption was that the difference was due to the inherently lower MC dose in bone and air, particularly on the periphery of the PTV, compared to the optimization SVD dose engine. Since bone and air are often encountered at the edge of the PTV, where the dose is already inherently lower, the renormalization required to obtain adequate target coverage would bring the hot spot up. While this effect is supposed to be mitigated by periodic full dose calculations during optimization, it may not be completely eliminated. However, this hypothesis was not borne out by the results. MC dose in bone is higher than DMSVD one, as shown by a simple slab phantom simulation, and yet switching the optimization dose algorithm to DMSVD did not help with improving target coverage and PTV dose homogeneity (Tables 2 and 4). At this point, we cannot provide a rigorous explanation for the remainder of the difference. Our results are qualitatively in agreement with Kamaledin et al.^11^ for another pair of Type B versus C algorithms; when *reoptimized* using the same objectives, their nasopharyngeal GBBR‐based plans exhibited worse target dose homogeneity and conformality compared to AAA.

**TABLE 4:** Analysis of variance (ANOVA) test of the maximum dose to PTV_High (*D*_0.03cc_) computed with different algorithms

	*D*_0.03cc_ (% of *Rx*)				
Descriptive statistics (*n* = 21)	Pinn‐CC	MC‐Recalc	RS‐CC	SVD‐MC	DMSVD‐MC
Median	104.9	107.4	105.1	106.0	106.1
Mean	104.8	107.6	105.0	106.0	106.1
SD	0.45	1.41	0.42	1.24	0.89
Lower 95% CI	104.6	107.0	104.8	105.5	105.7
Upper 95% CI	105.0	108.2	105.2	106.6	106.5

	Multiple pair‐wise comparisons *p*‐values				
MC‐Recalc	<0.0001				
RS‐CC	0.8888	<0.0001			
SVD‐MC	<0.0001	<0.0001	0.0009		
DMSVD‐MC	<0.0001	<0.0001	0.0005	0.9998	

Abbreviations: CC, collapsed cone; CI, confidence interval; MC, Monte Carlo; RS, RayStation; SVD, singular value decomposition.

For consistency, we used the same DVH evaluation software for the dose distributions generated by the two planning systems. It is also useful to know how the dose metrics would vary if the native TPS tools were used. To that end, we evaluated the *D*_0.03cc_ extracted by three different tools (two TPSs and ProKnow) from the dose distributions that could be evaluated by all three of them, namely, the original Pinnacle plans. The average *D*_0.03cc_ was 104.9 ± 0.5%, 104.9 ± 0.4%, and 105.2 ± 0.5% for ProKnow, Pinnacle, and RS tools, respectively. The differences are minimal and for the RS the error is in the safe direction—if anything, it may slightly overestimate the hot spot.

Given the relatively small but real and persistent increase in the hot spot discussed above, the obvious question was how to use the MC‐based planning tool clinically without sacrificing continuity of care. Looking at Table 4, the Pinnacle plans recalculated “as is” with the MC algorithm show the mean hot spot *D*_0.03cc_ 2.8% higher than the original (Pinnacle) one. The discrepancy between different TPS with their unique approaches to beam modeling and heterogeneity handling is expected. Since the original plans resulted in favorable clinical outcomes, it was considered a viable transition strategy to aim the MC planning for the hot spot values within the 95% confidence interval (CI) of the Pinnacle plans directly recalculated with RS MC. That meant putting the upper limit of 108% on the *D*_0.03cc_ hot spot (Table 4). Further examining the 95% CI for the plans optimized in RS with the MC final dose calculation suggested that a lower ∼107% hot spot should be achievable in most circumstances. While this is still higher than nominal 105% we were used to, in comparison with the Pinnacle plans recalculated with MC, it can be actually considered an improvement. Even if taken at face value, it is still substantially more stringent than the values accepted by the cooperative groups^43^, ^44^ and typically reported in the literature.^11^, ^13^, ^16^, ^49^, ^52^, ^53^ For broader comparison, we calculated the often used homogeneity index (HI) $HI\;=\left(D_{2\%}-D_{98\%}\right)/D_{50\%}\;$ for our patient cohort optimized with MC. Our average *HI* was 0.05 ± 0.01, or one‐half of the typically reported values for HN plans generated with various algorithms.^11^, ^16^, ^52^

Despite the best effort, it was virtually impossible to achieve the 105% *D*_0.03cc_ hot spot with MC in the vast majority of the cases. By the same token, it seldom exceeded 107%, although technically we still considered 108% acceptable. Finally, the current study showed a trend, albeit not rising to the statistical significancy level, of a slightly higher OAR doses with MC. This is consistent with the higher hot spot and VoR observed in MC plans. In this study, we kept the planning paramters as close as possible to the original ones, in particulalr using only two VMAT arcs. With RS, it is practical to routinely use three or more arcs since the planning time with MC is only a weak function of the number of beams. Giving the optimizer more degrees of freedom helps with plan quality in terms of OAR sparing.

## 5 CONCLUSIONS

When the hot spot in HN MC‐based treatment planning is defined at the *D*_0.03cc_ level, it is exceedingly difficult to consistently limit it to 105% of the prescription dose, as we were used to with the CC convolution algorithm. The average calculated hot spot after optimization and calculation with RS MC was statistically significantly higher compared to Pinnacle and RS CC algorithms by 1.2 and 1.0 %, respectively. Only a very small portion of this difference can be attributed to the statistical blurring of the target DVH. The 95% CI observed in this study suggests, however, that in most cases a hot spot of ≤107% is achievable. Compared to the 95% CI for the previous clinical plans recalculated with RS MC “as is” (upper limit 108%), in real terms this result is at least as good or better than the previous plans. It is feasible to transition to the presumably more accurate algorithm without sacrificing realistic target dose homogeneity. The difference in dose‐to‐bone was less than for other reported combination of Type B and C algorithms. The dose to soft tissue was essentially unaffected. When making a clinical transition, there is no generic “Type B to C” dosimetric recipe and the effect of the underlying algorithms and implementations need to be examined in detail.

## CONFLICT OF INTEREST

Agnes Angerud and Max Isacson are employees of RaySearch. The other authors declare no conflict of interest.

## AUTHOR CONTRIBUTIONS

Overall project design, data analysis, statistics, manuscript drafting, and approval: Vladimir Feygelman. Data selection and preparation, study design, planning, manuscript preparation, and approval: Kujtim Latifi. Study design, treatment planning, practice analysis, manuscript preparation, and approval: Mark Bowers and Kevin Greco. Overall project design, data evaluation, manuscript preparation, and approval: Eduardo Moros. Study design, software design, data analysis, manuscript preparation, and approval: Max Iscason and Agnes Angerud. Design of the clinical aspects of the study, patient selection, clinical evaluation, manuscript preparation, and approval: Jimmy Caudell.

## Supporting information



Supporting information

## Data Availability

Tabulated statistical data are available from the authors upon reasonable request. The original datasets cannot be made available.
